# The Effects of Superimposed Whole-Body Electromyostimulation During Short-Term Strength Training on Physical Fitness in Physically Active Females: A Randomized Controlled Trial

**DOI:** 10.3389/fphys.2019.00728

**Published:** 2019-06-27

**Authors:** Ulrike Dörmann, Nicolas Wirtz, Florian Micke, Mareike Morat, Heinz Kleinöder, Lars Donath

**Affiliations:** Department of Intervention Research in Exercise Training, Institute of Training Science and Sport Informatics, German Sport University Cologne, Cologne, Germany

**Keywords:** linear sprint, change of direction speed, electrical stimulation, power, isoinertial, plyometrics

## Abstract

The aim of this study was to compare the effects of short-term strength training with and without superimposed whole-body electromyostimulation (WB-EMS) on straight sprinting speed (SSS), change of direction speed (CODS), vertical and horizontal jumping, as well as on strength and power in physically active females. Twenty-two active female participants (*n* = 22; mean ± SD: age: 20.5 ± 2.3 years; height: 171.9 ± 5.5 cm; body mass: 64.0 ± 8.2 kg; strength training experience 5.1 ± 3.6 years) were randomly assigned to two groups: strength training (S) or strength training with superimposed WB-EMS (S+E). Both groups trained twice a week over a period of 4 weeks and differed in the application of free weights or WB-EMS during four strength (e.g., split squats, glute-ham raises) and five sprinting and jumping exercises (e.g., side and box jumps, skippings). The WB-EMS impulse intensity was adjusted to 70% of individual maximal sustainable pain. SSS was tested *via* 30-m sprinting, CODS by a T-run, vertical and horizontal jumping using four different jump tests at pre-, post-, and retests. Maximal strength (F_max_) and power (P_max_) testing procedures were conducted on the Leg Press (LP), Leg Extension (LE), and Leg Curl (LC) machine. Significant time × group interaction effects revealed significant decreases of contact time of the Drop Jump and split time of CODS (*p* ≤ 0.043; $\eta_{p}^{2}$ = 0.15–0.25) for S (≤ 11.6%) compared to S+E (≤ 5.7%). Significant time effects (*p* < 0.024; $\eta_{p}^{2}$ = 0.17–0.57) were observed in both groups for SSS (S+E: ≤6.3%; S: ≤8.0%) and CODS (S+E: ≤1.8%; S: ≤2.0%) at retest, for jump test performances (S+E: ≤13.2%; S: ≤9.2%) as well as F_max_ and P_max_ for LE (S+E: ≤13.5%; S: ≤13.3%) and LC (S+E: ≤18.2%; S: ≤26.7%) at post- and retests. The findings of this study indicate comparable effects of short-term strength training with and without superimposed WB-EMS on physical fitness in physically active females. Therefore, WB-EMS training could serve as a reasonable but not superior alternative to classic training regimes in female exercisers.

## Introduction

The importance of resistance training in order to enhance sprinting and jumping performance is generally accepted. The relevance of maximal strength and power training has been repeatedly underlined, especially in competitive sports for women and men (Reilly, 2007; Sander et al., 2013; Stojanović et al., 2017; Sommi et al., 2018). Sprinting and jumping performance such as straight sprinting speed (SSS) and change of direction speed (CODS) as well as vertical (VJ) and horizontal jumping (HJ) are basic abilities for successful participation in a variety of sports (Sheppard and Young, 2006; Young, 2006; Brughelli et al., 2008; Perez-Gomez and Calbet, 2013). The lack of incorporating such strength and conditioning approaches into training routines in female athletes compared with their male counterparts is still apparent (Sommi et al., 2018). The transfer of well-developed strength and power variables on sport-specific movement patterns is mostly not as clear as presumed (Sheppard and Young, 2006; Young, 2006; Brughelli et al., 2008). Relatively large gains in strength and power output can lead to meaningful increases in jumping but considerably less in sprinting performance (Young, 2006; Brughelli et al., 2008). Therefore, Young (2006) and Brughelli et al. (2008) highlighted the role of specific exercise movement patterns and contraction velocities in strength training exercises. Additionally, they recommended to perform plyometrics, horizontal jumps, lateral jumps, and loaded vertical jump training including bilateral and unilateral exercises as well as SSS- and CODS-specific skill training in order to adhere to specific movement requirements and directions to develop sprinting performance.

Electromyostimulation (EMS) is known as an effective complementary training method to improve athletic performance surrogates (Filipovic et al., 2011). The application of EMS beneficially affects several physiological pathways that induce adaptations: A higher number of motor units are recruited during exercises with superimposed EMS compared with dynamic voluntary contractions (VCs) alone (Kots and Chwilon, 1971). EMS activates fast-twitch fibers at relatively low force levels (Gregory and Bickel, 2005), and squat exercise with superimposed EMS can potentially induce an increase in recruitment of high-threshold motor units (Dudley, 1992). Moreover, EMS increases activation levels at different muscle length and during different contraction modes, e.g., during eccentric work phases (Willoughby and Simpson, 1998) and possibly reduces the difficulty to achieve sport-specific movement velocities within resistance training (Young, 2006) by a higher firing rate and a synchronization of motor units (Gregory and Bickel, 2005). Further advantages could be achieved by whole-body EMS (WB-EMS) devices that are able to stimulate several muscle groups simultaneously, e.g., muscle chains or agonist/antagonist during a multi-joint movement. WB-EMS triggers a counterproductive firing of the agonist and antagonist. This requires voluntary contractions to reduce co-activation of antagonistic muscles, in order to continue the required dynamic exercise (Wirtz et al., 2016). Most recent evidence suggests that EMS, superimposed onto VCs in a submaximal task, could result in greater muscle fibers recruitment than voluntary or electrical stimulation alone and would be likely to generate greater gains of motor output after a training period (Paillard, 2018). A low voluntary movement control exists at maximum stimulation intensities (Babault et al., 2007), and only submaximal contractions enable an efficient movement control with superimposed EMS (Bezerra et al., 2011). Submaximal dynamic WB-EMS is in accordance with the guidelines for a safe and effective WB-EMS training (Kemmler et al., 2016a). These guidelines consider that WB-EMS features many factors known to be associated with muscle damage, due to the ability to innervate large muscle areas simultaneously with individually tailored intensity per muscle group. For example, Malnick et al. (2016) reported diagnosis of rhabdomyolysis if EMS training was supervised inadequately. Due to the aforementioned background, the question arises whether submaximal WB-EMS during dynamic strength and/or sport-specific exercises over the entire muscle length and muscle chains leads to greater improvements in both jumping and sprinting as well as strength and power performance.

Up to now, there is a lack of studies dealing with submaximal superimposed WB-EMS on sprinting and jumping performance, especially using dynamic strength exercises in combination with jump-, SSS-, or CODS-specific skill training. Most interestingly, there are no available WB-EMS studies in female athletes. Only two studies dealt with the transfer into sprinting and jumping performance with male athletes (Filipovic et al., 2016; Wirtz et al., 2016). Both used squat exercises with superimposed WB-EMS as a strength training intervention. No further exercises for sprinting or jumping performance were conducted. Both studies enhanced CODS (2.4 and 5.5%) and jumping performance (8.1 and 8.7%). SSS only increased for the WB-EMS group in comparison with the control group during 14-week intervention with elite soccer players at 5-m split time (2.9%) (Filipovic et al., 2016).

Therefore, the aim of this study was to compare the effects of short-term strength training with and without superimposed WB-EMS on (1) SSS and CODS, on VJ and HJ, as well as on (2) strength and power parameters in female strength trained sport students. It was hypothesized that short-term strength training with submaximal superimposed WB-EMS improves physical fitness in physically active females more than short-term strength training without superimposed WB-EMS.

## Materials and Methods

### Study Design

**FIGURE 1:**
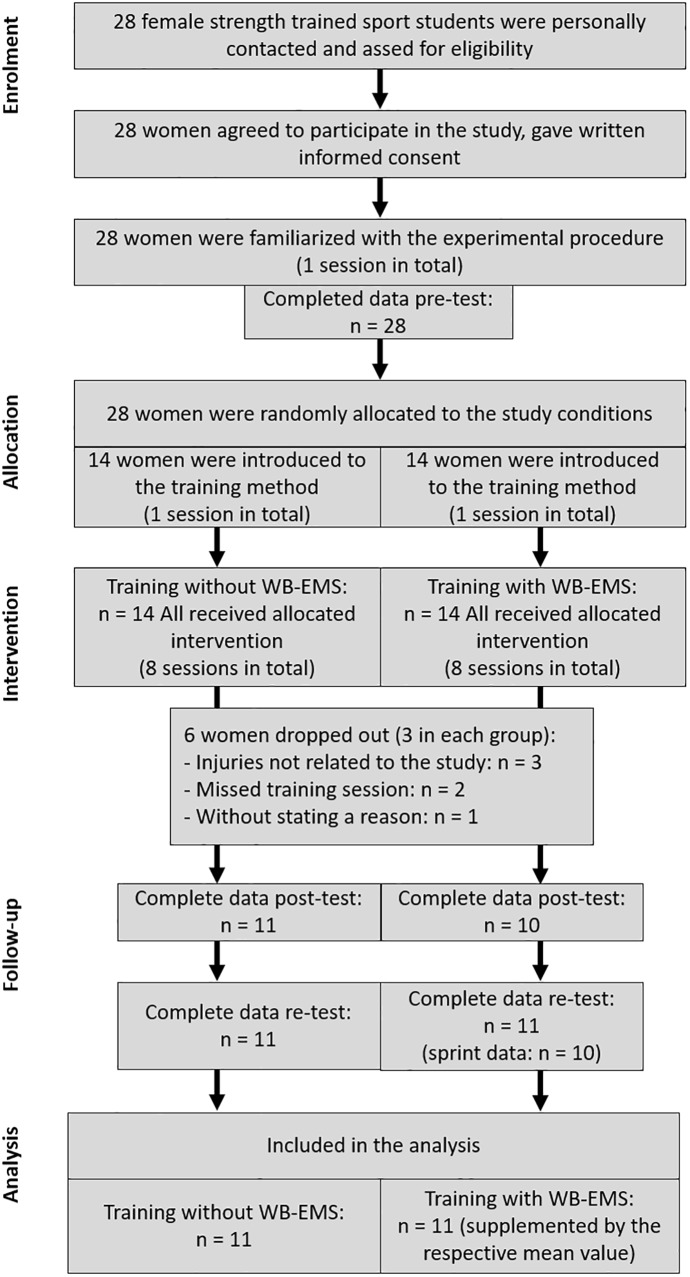
Participants flow through the study [adapted from Moher et al. (2010)].

This study was designed as a two-armed randomized controlled trial with a parallel-group design comparing effects of submaximal, superimposed dynamic WB-EMS (S+E) with effects of dynamic athletic training without WB-EMS (S) on sprinting and jumping performance as well as on strength and power. S+E and S completed eight training sessions in 4 weeks. To determine training effects, the sprint, jump, strength, and power diagnostics were intra-individually conducted on three occasions at the same time of the day under constant and stable lab conditions: directly before, directly after the training period (pre- and post-tests), and after a 2-week follow-up (retest) (Figure 1). The timing of the retest was derived from several EMS studies with delayed strength adaptations after a detraining period of 2–6 weeks (Filipovic et al., 2011). After pretesting, the subjects were randomly assigned to either S+E or S. In order to minimize influences of unspecific training loads, both groups were asked to refrain from any changes of their habitual physical activity behavior. Furthermore, all participants were instructed to maintain their normal dietary intake before and during the study.

### Participants

Twenty-eight female strength trained sport students participated in the study. According to Araújo and Scharhag (2016), they can be classified as exercisers and not as athletes. They were medically examined with regard to the musculoskeletal and the cardiovascular systems (exercise electrocardiogram) and signed a consent document about the possible risks and benefits of the study. Exclusion criteria were planned absences during the whole study period, a missed training session, any training experience in WB-EMS, current training programs focusing on sprinting and jumping, as well as inadequate technique in the strength exercises used. One week before the pretests, the participants were familiarized with the experimental procedure. Jump and strength diagnostics were practiced in a sample session until the participants showed a technically correct execution in jumping as well as a variation between the trials smaller or equal to the typical error examined in test–retest procedures of our strength diagnostics lab. After the randomization, the training methods were introduced in a sample session (Figure 1). It also included the verification of the individual eight to 10 repetition maximum for the strength exercises for S as well as fitting of the electrodes and familiarization to the electrical stimulus during the exercises for S+E. Six participants, three in each group, terminated their participation. Three suffered injuries that were not related to the study. Two missed a training session. One left the study at her own request without stating a reason. Finally, 22 participants completed all training sessions and were included in the analysis of the results with a 100% attendance rate for both groups (*n* = 22; participants characteristics are presented in Table 1). One person from S+E failed the complete posttest. One person from S+E was absent for the sprint testing at retest. The data were supplemented by the respective mean value of the group. The study protocol was approved by the Ethics Committee of the German Sport University Cologne in December 2013 and complied with the Declaration of Helsinki.

**Table 1:** Anthropometric data (mean ± SD).

	N	Age (years)	Height (cm)	Weight (kg)	BMI (kg/m^2^)	Strength Training Experience (years)
S+E	11	20.4 ± 2.8	172.7 ± 7.3	65.5 ± 10.7	21.8 ± 2.4	6.5 ± 3.9
S	11	20.5 ± 1.8	170.3 ± 3.8	62.0 ± 4.7	21.4 ± 1.6	3.9 ± 3.2

### Training Procedure

Both groups completed eight training sessions over a 4-week period. The length of the training period and the number of training sessions were derived from numerous EMS studies with an average period of 4–6 weeks with one to seven sessions per week (Filipovic et al., 2012). The two sessions per week were methodologically different. One was focused on strength exercises lasting 25 min and one on jumping and sprinting exercises lasting 20 min. The participants of both groups similarly performed the training sessions with the only difference that S+E performed all exercises superimposed by WB-EMS and S the strength exercises with additional loads (ALs).

The WB-EMS intervention complied with the guidelines for a safe and effective WB-EMS training (Kemmler et al., 2016a). The miha bodytec system (Augsburg, Germany) was selected as EMS device (Kemmler et al., 2012; van Buuren et al., 2013). It was an application unit that was connected *via* electrical cords to a stimulation vest and belts (see Supplementary Figure S1). Bilaterally paired surface electrodes were integrated. Thus, eight muscle areas could be stimulated synchronously with freely selectable impulse intensities (0–120 mA) for each pair of electrodes. In our study, three paired electrodes were applied around the muscle belly of the lower legs (27 cm length × 4 cm width), the thighs (44 × 4 cm) and at the buttocks (13 × 10 cm). Additionally, the upper body was stimulated with two bilaterally paired electrodes that were integrated in the stimulation vest at the lower back (14 × 11 cm) and at the abdominal (23 × 10 cm). This simultaneous stimulation was used for all exercises, and the application of the electrodes took 5 min for each S+E participant before each session.

The intensity of WB-EMS was adjusted to 70% of the individual pain threshold (iPT = maximum tolerated amperage, 0–120 mA). The iPT was verified separately for each pair of electrodes before each session and lasted 2 min for each S+E participant. The participants stood with an interior knee angle of 170° while tensing their lower limbs muscles. The verification of iPT began with the electrodes at the buttock, followed by the thigh, the lower leg, the abdominal, and the lower back electrodes. Then, the intensity was subsequently downregulated with the main controller at the WB-EMS device to an intensity of 70% to enable dynamic movements. The impulse frequency was set at 85 Hz, the impulse width at 350 μs, the impulse type as bipolar and rectangle (Filipovic et al., 2016; Kemmler et al., 2016b; Wirtz et al., 2016). On/off-time was adjusted for each exercise (see Tables 2, 3). In general, EMS was applied during all the execution time of each exercise and stopped during the rest period.

**Table 2:** Strength training exercises with characteristics about repetitions, sets, rest between the sets, contraction mode (ecc = eccentric, iso = isometric, con = concentric) per repetition, range of motion per repetition and time under tension (TUT) per exercise for both groups as well as on/off ratio of WB-EMS impulse (70% of the individual pain threshold) for the strength training group with superimposed WB-EMS (S+E) and additional load (individual 8–10 repetition maximum) for the strength training group (S).

	S+E Group and S Group						S+E Group	S Group
Strength Training Exercises	Repetition (n)	Set (n)	Rest (s)	ECC: ISO: CON (s)	ROM^#^ (°)	TUT (s)	On/Off Ratio (s)	Additional Load
(1) Bulgarian Split Squat	10	3 per leg^∗^	60	2: 1: 2	170–90	300	50/5	≤20 kg Barbells
(2) Nordic Curl	8	3	60	2: 0: 2	90–135	96	32/0	Softer Rubber Band
(3) Knee Tuck	8	3	60	2: 0: 2	180–70	96	32/0	–
(4) Side Abs	8 per side	3	60	0.5: 0: 0.5	–	48	16/0	≤4 kg Medicine Ball

*^#^Range of motion (ROM) of the interior knee or interior hip angle. ^∗^The front position of the legs was changed alternately after each set.*

**Table 3:** Jumping and sprinting training exercises with characteristics about repetitions, sets, rest between the sets, range of motion per repetition and time under tension (TUT) per exercise for both groups as well as on/off ratio of WB-EMS impulse (70% of the individual pain threshold) for the strength training group with superimposed WB-EMS (S+E) and additional load (individual 8–10 repetition maximum) for the strength training group (S).

	S+E Group and S Group					S+E Group	S Group
Jumping and Sprinting Training Exercises	Repetition or Duration (n or s)	Set (n)	Rest (s)	ROM^#^ (°)	TUT (s)	On/Off-Ratio (s)	Additional Load
(1) Skipping	8 s	3	30	180–90	24	8/0	–
(2) Heeling	10	3 per leg^∗^	30	180–90	60	10/5	–
(3) Side Jump	5 per side	3	30	–	36	12/0	–
(4) Box Jump	5	3 per leg^∗^	30	–	90	3/5	–
(4) Drop Jump	5	3	30	–	45	3/5	–

*^#^Range of motion (ROM) of the interior hip angle. ^∗^Legs were changed alternately after each set.*

The strength sessions involved four exercises for both groups: (1) Bulgarian split squat: single leg split squat with heel raise, elevated rear foot, and hands remaining in the akimbo position, (2) Nordic curl: two-legged hamstring curl with supporting rubber bands at chest height, (3) knee tuck: knee pull to the chest while feet hang in loops and upper body remain in push-up position, as well as (4) side abs: side-to-side medicine ball crunch with raised legs (exercise characteristics are presented in Table 2; pictures of each exercise are presented in the Supplementary Table S1).

During each exercise, temporal distribution of contraction modes was standardized per repetition by an acoustical signal at start and end positions of the exercise. The intensity of each exercise set was controlled by Borg rating of perceived exertion (RPE) (Tiggemann et al., 2010). If a set was no longer exhaustive (RPE < 16 “hard”), the impulse intensity was raised for S+E during the training period. S enhanced the intensity for (1) Bulgarian split squat by adding free weights ≤20 kg, for (2) Nordic curl by adding softer rubber bands (gold to black, Gymstick; Ludwig Artzt GmbH, Dornburg, Germany), and for (4) side abs by adding a heavier medicine ball (Fitness-Mad, Worcestershire, United Kingdom; ≤4 kg). A TRX (Fitness Anywhere, San Francisco, United States) was used as sling system for (3) knee tucks. The intensity could have been increased by stabilizing only one instead of two legs in the TRX. There was no need for this variation during the training period (RPE > 16).

The sessions focusing on sprinting and jumping involved five exercises for both groups: (1) skipping: knee lever runs against a rubber band fixed around the hips, (2) heeling: single leg heels with fore-swinging of the lower leg and active foot attachment when returning the swing leg and hands remaining in the akimbo position, (3) side jumps: single leg side jump from one leg to the other about a 20-cm hurdle, (4) box jumps: single leg box jump after a two-step start on a 38-cm box with 1-m jump distance, as well as (5) drop jumps: drop jumps from a 38-cm box with hands remaining in the akimbo position (exercise characteristics are presented in Table 3, pictures of each exercise are presented in the Supplementary Table S2). These exercises followed the recommendations of Young (2006), Brughelli et al. (2008), and Stojanović et al. (2017) to increase SSS and CODS as well as VJ and HJ. The intensity of each sprinting and jumping exercise set was controlled by RPE, too. Both groups enhanced the intensity by a maximum movement frequency or an explosive movement speed. The impulse intensity for S+E had been adjusted at 70% of the iPT.

The warm-up consisted of 5-min cycling before each session. The rest between the exercises was 2.5 min. Thus, the total contact time for S lasted 30 min for the strength sessions as well as 25 min for the jumping and sprinting sessions. S+E had a 7-min (5-min application of the electrodes plus 2-min verification of impulse intensity) longer total contact time for each session.

### Testing Procedure

#### Sprint Testing

**FIGURE 2:**
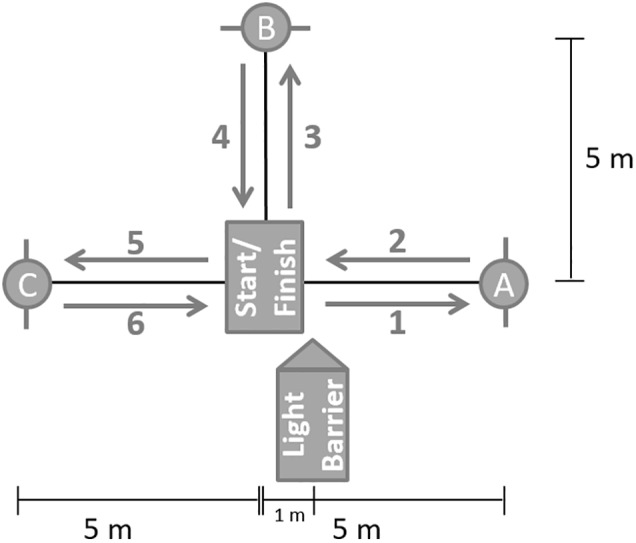
T-run: Cone A, B, and C had to be reached after one another by a forward sprint (1–5). Marks on the floor must be crossed at each corner. After cone C (6), participants could run as fast as possible across the finish line [adapted from McBride et al. (2002) and Reiman and Manske (2009)].

Sprint testing involved a T-run for CODS (Figure 2) and a 30-m sprint for SSS. Both tests were performed with a self-initiated standing start with no hopping or backward movement before the start. The split times were measured for 30-m sprint at 5, 10, and 20 m. The split time values for T-run were measured after the first change of direction at cone A (Figure 2). Double infrared photoelectric barriers (DLS/F03, Sportronic, Leutenbach-Nellmersbach, Germany) were used to measure time. The sprinting time as the best of two attempts was used for subsequent analysis. The participants had 2-min rest between the trials.

Moreover, a tapping test about 5 s was conducted with the OptoJump system (Microgate, Bolzano, Italy). It is based on measurements of optical light emitting diodes. The participants were instructed to complete as many steps as possible in 5 s and to start on their own. The system automatically counted the total number of steps for 5 s from the first step. The parameter as the best of two trials was total steps (n). The participants had 2 min rest between the trials.

#### Jump Testing

After one familiarization jump trial, the participants performed three trials of each jump variation in a fixed order: (1) standing long jump (SLJ), (2) counter movement jump (CMJ), (3) squat jump (SJ), as well as (4) drop jump (DJ). For the SLJ (1), the participants were instructed to start jumping from an upright standing position, squatting down to an adequate momentum in order to jump as long as possible. The jump distance was measured from the start line to the participants’ heel. The attempts were invalid if the participant stepped back or forward after landing. For the CMJ (2), participants were instructed to start jumping from an upright standing position, squatting down to a knee angle of approximately 90° in order to jump as high as possible. For the SJ (3), participants were instructed to start jumping from a static semi-squatted position holding the knees at 90° without any preliminary movement. The DJ (4) started from a 38-cm box. The participants were instructed to drop down from the box and then to jump as high as possible after a short contact time on the ground. The OptoJump system (Microgate, Bolzano, Italy) was used to verify jump height and contact time using the flight time method. Thereby, hands remained in the akimbo position for the entire movement of each jump to minimize the influence of arm swing. The jump with the greatest height or distance for each variation was subsequently used for analysis. The DJ performance was evaluated by the highest reactive strength index (RSI) (Flanagan and Comyns, 2008; Prieske et al., 2018; Ramirez-Campillo et al., 2018), which was calculated by dividing the jump height by the corresponding ground contact time (Healy et al., 2018).

#### Strength and Power Testing

Strength and power diagnostics took place on the leg curl (LC), the leg extension (LE), and the leg press (LP) machine (Edition-Line, gym80, Gelsenkirchen, Germany). Those were equipped with the digital measurement equipment Digimax (mechaTronic, Hamm, Germany). It enabled the measurement of the peak force F_max_ and the peak power P_max_ (5-kN strength sensor type KM1506, distance sensor type S501D, megaTron, Munich, Germany) employing the software IsoTest and DynamicTest 2.0. The sensors were installed in line with the steel belt of the machines that lifts the selected weight plates.

Diagnostic procedures consisted of three isometric trials for each machine. Isometric attempts were conducted at an interior knee angle of 120° for LE and LP as well as of 150° for LC. The instruction was to press as forcefully and as fast as possible against the fixed lever arm. This enabled to determine knee joint angle-dependent force–time curve during explosive maximum VC and to calculate the parameter F_max_ (N) as the isometric peak force. Moreover, diagnostic procedures consisted of six isoinertial trials for LE and LC as well as three isoinertial trials for LP. The isoinertial test attempts were conducted with an additional load (AL). AL was individually calculated as a percentage of F_max_ in a further isometric test with the same angle as the starting position of the isoinertial test (LE and LP 90°; LC 170°). Three attempts were conducted with 40% AL for LE and LC as well as three attempts with 60% AL for LP, LE, and LC. Concerning isoinertial tests, the participants were introduced to move the lever arm as forcefully and as fast as possible over the complete concentric range of motion. This enabled to examine knee joint angle-dependent power–load curve during explosive maximum voluntary LE for LP, knee extension for LE, or knee flexion for LC and to calculate the parameter P_max_ as the concentric dynamic peak power. The concentric range of motion corresponded to 90–180° for LP and LE as well as to 170–80° for LC (interior knee angle). The rest design was 60 s between the trials and 3 min between the strength machines, respectively. The parameters F_max_ (N) and P_max_ (W) were calculated for statistical analysis and data presentation as the best of three trials.

### Statistical Analysis

The data of the 22 participants were reported as mean value ± SD. All data were normally distributed for all groups except for F_max_ for LC at posttest (*p* = 0.010), P_max_ with 40% AL for LE at retest (*p* = 0.050), P_max_ with 60% AL for LP (*p* = 0.033), SSS 10-m split time (*p* = 0.032) and SJ (*p* = 0.002) at pretest for S+E as well as P_max_ with 40% for LC at pretest (*p* = 0.050) for S, as assessed by Kolmogorov–Smirnov test (*p* > 0.05). With a closer look at the quantile–quantile plots and according to Berkovits et al. (2000), the data were continued without applying measures. There was homogeneity of the error variances, as assessed by Levene test (*p* > 0.05). To determine the effect of the training interventions, a separate 3 × 2 (time × group)-mixed ANOVA with repeated measures was conducted. ANOVA assumption of homogenous variances was tested using Maulchy test of sphericity. If a violation of Mauchly’s test was observed, Greenhouse–Geisser correction was used. Partial eta-square ($\eta_{p}^{2}$) values were reported as effect size for significant main effects of the ANOVA with $\eta_{p}^{2}$ ≥ 0.01 indicating small, ≥ 0.059 medium, and ≥ 0.138 large effects (Cohen, 1988). If 3 × 2-mixed ANOVA revealed a significant time or time × group interaction effect on any variable, this effect was further investigated carrying out Bonferroni-corrected *post hoc* pairwise comparison (*p* < 0.05). In this context, standardized mean differences (SMD) were calculated between pre-, post-, and retests. Thresholds for small, moderate, and large effects were 0.20, 0.50, and 0.80, respectively (Cohen, 1988). SPSS 25.0 (IBM^®^, Armonk, NY, United States) was used for all statistical procedures.

Reliability was determined by the coefficient of variation (CV) and the intraclass correlation coefficient (ICC) for F_max_ (CV < 8%; ICC 0.95–0.97) and for P_max_ (CV < 9%; ICC 0.84–0.97) during a week-long test–retest procedure. Previously, measures of CODS, SSS, and jump performance have been shown to be highly reliable (CV 1–9%; ICC 0.80–0.99) (Markovic et al., 2004; Brughelli et al., 2008; Casartelli et al., 2010; Glatthorn et al., 2011; Green et al., 2011; Ball and Zanetti, 2012).

## Results

### Sprint Testing

Sprint values for both groups are provided in Table 4. A statistically significant and large interaction between time × group was observed for split time of CODS (*p* = 0.002; $\eta_{p}^{2}$ = 0.25). Only S showed a significantly higher performance for T-run split time between pre- and retests following *post hoc* analyses (*p* = 0.001).

**Table 4:** Changes in 30-m linear sprint (LS) and in T-run for total time (TT) and split time (ST) as well as in tapping test for total steps in group S (strength training) and S+E (strength training with superimposed WB-EMS) during pre-, post-, and retests.

	Parameter	Group	Pretest	Posttest	Pre–Post		Retest	Pre–Re		ANOVA p ($\eta_{p}^{2}$)		
					% Delta	SMD		% Delta	SMD	Time	Group	Time^∗^ Group
Straight Sprinting Speed	LS TT	S+E	4.80 0.23	4.75 0.19	–1.0	0.24	4.69 0.24	−2.3^∗^	0.47	**<0.001 (0.323)**	0.336 (0.046)	0.954 (0.002)
					
	(s)	S	4.73 0.20	4.67 0.21	−1.3	0.29	4.60 0.21	−2.8^∗^	**0.63**			
				
	LS 5-m ST	S+E	1.11 0.05	1.09 0.04	−1.8	0.44	1.04 0.05	−6.3^∗^	**1.40**	**<0.001 (0.548)**	0.666 (0.009)	0.620 (0.024)
					
	(s)	S	1.12 0.05	1.07 0.06	−4.5	**0.91**	1.03 0.03	−8.0^∗^	**2.18**			
				
	LS 10-m ST	S+E	1.92 0.10	1.92 0.07	0.0	0.0	1.85 0.09	−3.7^∗^	**0.74**	**<0.001 (0.480)**	0.527 (0.020)	0.705 (0.013)
					
	(s)	S	1.92 0.07	1.90 0.09	−1.0	0.25	1.82 0.07	−5.2^∗^	**1.43**			
				
	LS 20-m ST	S+E	3.40 0.16	3.37 0.12	−0.9	0.21	3.30 0.17	−2.9^∗^	**0.61**	**<0.001 (0.408)**	0.392 (0.037)	0.863 (0.007)
					
	(s)	S	3.36 0.13	3.32 0.15	−1.2	0.29	3.24 0.13	−3.6^∗^	**0.92**			

Tappings	Total Steps in 5 s	S+E	45.00 6.05	46.82 5.31	+4.0	0.32	49.27 5.29	+9.5^∗^	**0.75**	**0.005 (0.306)**	0.419 (0.033)	0.506 (0.025)
					
	(n)	S	45.09 8.41	49.18 4.45	+9.1	**0.61**	51.64 3.85	+14.5^∗^	**1.00**			

Change of Direction Speed	T-run TT	S+E	8.72 0.36	8.67 0.25	−0.6	0.16	8.56 0.33	−1.8^∗^	0.46	**0.008 (0.215)**	0.426 (0.032)	0.816 (0.010)
					
	(s)	S	8.62 0.33	8.62 0.23	0.0	0.00	8.45 0.19	−2.0^∗^	**0.63**			
				
	T-run ST	S+E	2.40 0.11	2.35 0.09	−2.1	**0.50**	2.39 0.12	−0.4	0.09	**0.024 (0.170)**	0.400 (0.036)	0.002 (0.247)
					
	(s)	S	2.38 0.10	2.39 0.06	+0.4	0.12	2.28 0.06°	−4.2	**1.21**			

*Values are presented as mean ± SD. Moderate to large standardized mean differences (SMD) have been highlighted in bold. Significance level for time effects for both groups to pretest was set at p ≤ 0.05^∗^. Significance level for group^∗^time interaction effects was set at p ≤ 0.05°.*

Significant main effects of time were found for SSS for total time (*p* < 0.001; $\eta_{p}^{2}$ = 0.32), 5-m (*p* < 0.001; $\eta_{p}^{2}$ = 0.55), 10-m (*p* < 0.001; $\eta_{p}^{2}$ = 0.48), and 20-m split time (*p* < 0.001; $\eta_{p}^{2}$ = 0.41) as well as for CODS for total time (*p* = 0.008; $\eta_{p}^{2}$ = 0.22). Total steps of tapping test showed significant main effects of time (*p* = 0.005; $\eta_{p}^{2}$ = 0.31), too. Bonferroni-adjusted *post hoc* analysis revealed a significant improvement for both groups between pre- and retests, respectively.

### Jump Testing

Jump values for both groups are provided in Table 5. There was a significant and large interaction between time × group for contact time of DJ (*p* = 0.043; $\eta_{p}^{2}$ = 0.15). Only S showed a significantly higher performance for contact time of DJ between pre- and posttests (*p* = 0.007) as well as pre- and retests (*p* = 0.004).

**Table 5:** Changes in standing long jump (SLJ), squat jump (SJ) and counter movement jump (CMJ), as well as in drop jump (DJ) for length, height, contact time, and reactive strength index (RSI) in group S (strength training) and S+E (strength training with superimposed WB-EMS) during pre-, post-, and retests.

	Parameter	Group	Pretest	Posttest	Pre–Post		Retest	Pre–Re		ANOVA p ($\eta_{p}^{2}$)		
					% Delta	SDM		% Delta	SDM	Time	Group	Time^∗^ Group
SLJ	Length	S+E	148.82 17.13	159.30 14.67	+7.0^∗^	**0.66**	158.64 13.24	+6.6^∗^	**0.64**	**<0.001 (0.574)**	0.333 (0.047)	0.145 (0.092)
					
	(cm)	S	155.55 8.78	161.55 14.71	+3.9^∗^	**0.50**	166.64 13.91	+7.1^∗^	**0.95**			

SJ	Height	S+E	25.25 3.79	27.17 3.59	+7.6	**0.52**	27.58 4.66	+9.2^∗^	**0.55**	**<0.001 (0.345)**	0.140 (0.106)	0.245 (0.068)
					
	(cm)	S	28.59 4.03	28.98 4.72	+1.4	0.09	30.46 4.65	+6.5^∗^	0.43			

CMJ	Height	S+E	27.36 3.83	28.89 3.14	+5.6^∗^	0.44	30.97 4.70	+13.2^∗^	**0.84**	**<0.001 (0.423)**	0.099 (0.130)	0.746 (0.015)
					
	(cm)	S	30.54 3.89	32.08 4.72	+5.0^∗^	0.36	33.36 5.05	+9.2^∗^	**0.63**			

DJ	Height	S+E	25.21 2.66	27.04 3.03	+7.3	**0.64**	27.53 3.35	+9.2	**0.77**	0.486 (0.035)	0.241 (0.068)	0.163 (0.087)
					
	(cm)	S	24.85 4.69	24.60 6.53	−1.0	0.01	24.21 5.34	−1.5	0.04			
				
	Contact Time	S+E	0.177 0.02	0.167 0.01	−5.7	**0.63**	0.177 0.02	+0.0	0.0	**0.001 (0.284)**	0.218 (0.075)	**0.043 (0.146)**
					
	(s)	S	0.199 0.03	0.176 0.02°	−11.6	**0.90**	0.178 0.03°	−10.6	**0.70**			
				
	RSI	S+E	1.46 0.31	1.63 0.24	+11.6^∗^	**0.61**	1.59 0.32	+8.9	0.41	**0.011 (0.202)**	0.149 (0.101)	0.969 (0.002)
					
		S	1.28 0.32	1.43 0.43^∗^	+11.7^∗^	0.40	1.39 0.35	+8.6	0.33			

*Values are presented as mean ± SD. Moderate to large standardized mean differences (SMD) have been highlighted in bold. Significance level for time effects for both groups to pretest was set at p ≤ 0.05^∗^. Significance level for group^∗^time interaction effects was set at p ≤ 0.05°.*

Significant main effects of time were observed for SLJ (*p* < 0.001; $\eta_{p}^{2}$ = 0.57), SJ (*p* < 0.001; $\eta_{p}^{2}$ = 0.35) and CMJ (*p* < 0.001; $\eta_{p}^{2}$ = 0.42), and RSI (*p* = 0.011; $\eta_{p}^{2}$ = 0.20). Bonferroni-adjusted *post hoc* analysis revealed a significantly higher SLJ and CMJ performance between pre- and posttests as well as pre- and retests for both groups. Both groups showed a significantly higher SJ performance between pre- and retests and a significantly higher RSI between pre- and posttests in the *post hoc* comparison.

### Strength and Power Testing

Strength and power values for both groups are provided in Tables 6, 7.

**Table 6:** Changes in maximal strength (F_max_) and power (P_max_) with 40 and 60% additional load for leg curl (LC) and leg extension (LE) in group S (strength training) and S+E (strength training with superimposed WB-EMS) during pre-, post-, and retests.

	Parameter	Group	Pretest	Posttest	Pre-Post		Retest	Pre-Re		ANOVA p ($\eta_{p}^{2}$)		
					% Delta	SDM		% Delta	SDM	Time	Group	Time^∗^ Group
LC	F_max_	S+E	725 116	843 177	+16.3^∗^	**0.79**	857 168	+18.2^∗^	**0.91**	**<0.001 (0.376)**	0.886 (0.001)	0.560 (0.029)
					
	(N)	S	722 243	859 161	+19.0^∗^	**0.67**	813 195	+12.6^∗^	0.41			
				
	P_max_ 40%	S+E	339 81	400 100	+18.0^∗^	**0.67**	392 94	+15.6^∗^	**0.60**	**<0.001 (0.564)**	0.910 (0.001)	0.139 (0.098)
					
	(W)	S	333 107	390 102	+17.1^∗^	**0.55**	422 103	+26.7^∗^	**0.85**			
				
	P_max_ 60%	S+E	405 81	445 94	+9.9^∗^	0.46	449 95	+10.9^∗^	**0.50**	**<0.001 (0.424)**	0.701 (0.008)	0.769 (0.013)
					
	(W)	S	382 98	433 106	+13.4^∗^	**0.50**	441 88	+15.5^∗^	**0.63**			

LE	F_max_	S+E	1507 202	1657 330	+10.0^∗^	**0.55**	1697 337	+12.6^∗^	**0.68**	**<0.001 (0.475)**	0.509 (0.022)	0.899 (0.005)
					
	(N)	S	1445 255	1566 277	+8.4^∗^	0.45	1622 258	+12.2^∗^	**0.69**			
				
	P_max_ 40%	S+E	691 150	768 171	+11.1^∗^	0.48	784 204	+13.5^∗^	**0.52**	**<0.001 (0.379)**	0.716 (0.007)	0.711 (0.017)
					
	(W)	S	717 193	812 199	+13.3^∗^	0.49	795 173	+10.9^∗^	0.43			
				
	P_max_ 60%	S+E	663 143	707 154	+6.6	0.30	684 145	+3.2	0.15	0.091 (0.113)	0.391 (0.037)	0.105 (0.106)
					
	(W)	S	714 185	720 131	+0.8	0.04	777 128	+8.8	0.40			

*Values are presented as mean ± SD. Moderate to large standardized mean differences (SMD) have been highlighted in bold. Significance level for time effects for both groups to pretest was set at p ≤ 0.05^∗^.*

**Table 7:** Changes in maximal strength (F_max_) and power (P_max_) with 60% additional load for leg press (LP) in group S (strength training) and S+E (strength training with superimposed WB-EMS) during pre-, post-, and retests.

	Parameter	Group	Pretest	Posttest	Pre–Post		Retest	Pre–Re		ANOVA p ($\eta_{p}^{2}$)		
					% Delta	SDM		% Delta	SDM	Time	Group	Time^∗^ Group
LP	F_max_	S+E	2905 565	3087 706	+6.3	0.29	2719 781	−6.4	0.27	0.127 (0.098)	0.315 (0.050)	0.546 (0.030)
					
	(N)	S	2616 656	2737 667	+4.6	0.18	2619 448	+0.1	0.01			
				
	P_max_ 60%	S+E	742 161	837 140	+12.8	**0.63**	804 158	+8.4^∗^	0.39	**0.010 (0.204)**	0.480 (0.025)	0.113 (0.103)
					
	(W)	S	820 250	837 231	+2.1	0.07	897 205	+9.4^∗^	0.34			

*Values are presented as mean ± SD. Moderate to large standardized mean differences (SMD) have been highlighted in bold. Significance level for time effects for both groups to pretest was set at p ≤ 0.05^∗^.*

Significant main effects of time were found for LC for F_max_ (*p* < 0.001; $\eta_{p}^{2}$ = 0.38), P_max_ with 40% (*p* < 0.001; $\eta_{p}^{2}$ = 0.56) and 60% (*p* < 0.001; $\eta_{p}^{2}$ = 0.42) AL, for LE for F_max_ (*p* < 0.001; $\eta_{p}^{2}$ = 0.48) and P_max_ with 40% AL (*p* < 0.001; $\eta_{p}^{2}$ = 0.38) as well as for LP for P_max_ with 60% AL (*p* = 0.010; $\eta_{p}^{2}$ = 0.20). The significantly higher performances were shown for both groups for LE and LC between pre- and posttests as well as pre- and retests in the *post hoc* comparison. The significant increases for LP exclusively occurred between pre- and retests.

## Discussion

This study compared the effects of short-term strength training with and without superimposed WB-EMS on (1) SSS and CODS, on VJ and HJ, as well as on (2) strength and power parameters in female strength trained sport students. It was hypothesized that short-term strength training with submaximal superimposed WB-EMS improves physical fitness in physically active females more than short-term strength training without superimposed WB-EMS.

There is a lack of studies dealing with submaximal superimposed WB-EMS on sprinting and jumping performance, especially using dynamic strength exercises in combination with sprinting and jumping exercises. Moreover, there are no available WB-EMS studies in female athletes.

Against the hypothesis, the findings of this study indicated no advantageous effects for short-term strength training in favor to submaximal superimposed WB-EMS (S+E) in comparison with strength training alone (S) on physical fitness in physically active females. Both groups, S as well as S+E, significantly increased the parameters F_max_ for LC and LE as well as P_max_ for LC, LE, and LP over time. Moreover, both groups transferred these strength and power gains into a significantly greater performance of the primary endpoints like total time of CODS, split and total time of SSS, as well as VJ height, RSI, and HJ length over time. Thus, both training methods, S and S+E, confirmed the results of existing meta-analyses in this context (Perez-Gomez and Calbet, 2013; Seitz et al., 2014). According to Young (2006) and Seitz et al. (2014), the transfer of the strength increases (S: 19.0%; S+E: 18.2%) into jumping performance (S: 11.7%; S+E: 13.2%) was higher than into sprinting performance (S: 2.8%; S+E: 2.3%), too. Moreover, the findings of S+E are in line with the two dynamic WB-EMS studies with males. They improved sprint time with change of directions by 5.5% at 15 m (Filipovic et al., 2016) and 2.4% at 30 m (Wirtz et al., 2016) as well as SJ performance by 8.1% (Filipovic et al., 2016) and 8.7% (Wirtz et al., 2016), respectively. Following Kots and Chwilon (1971), a higher number of motor units are recruited during exercises with superimposed EMS in comparison with dynamic VC alone. Moreover, EMS increases the activation levels at different muscle length and during different contraction modes, especially during eccentric work phases as Willoughby and Simpson (1998) hypothesized. According to Paillard (2018), EMS superimposed onto VCs in a submaximal task could result in greater muscle fibers recruitment than with voluntary stimulation alone. However, no greater gains of motor output could be generated in comparison with the present strength training regime (S) after the short-term training period, in particular for DJ ground contact time and CODS split time.

In contrast, the main findings of this study revealed a significant time × group interaction effect on split time of CODS and contact time of DJ for S in *post hoc* analysis. The split time significantly decreased at 5 m for S (4.2%) in comparison with S+E (0.4%) between pre- and retests (*p* < 0.002) and the contact time of DJ for S (11.6%; 10.6%) in comparison with S+E (5.7%; 0.0%) between pre-, post-, and retests (*p* < 0.043) in *post hoc* analysis. The two available WB-EMS studies investigated CODS at 15 m (Filipovic et al., 2016) and at 30 m (Wirtz et al., 2016) but no split time at 5 m. Moreover, Filipovic et al. (2016) analyzed RSI with positive effects for the WB-EMS intervention group but no height or contact time of DJ performance. Therefore, the present result cannot be placed in a larger context of WB-EMS. However, these initial results offer a practical recommendation. Concerning a large effect size (SMD = 1.21) for CODS at 5 m and a moderate to large effect size (SMD = 0.70–0.90) for contact time of DJ, dynamic strength training combined with jumping and sprinting exercises without superimposed WB-EMS (S) is currently to be preferred for physically active females. Perez-Gomez and Calbet (2013) reviewed training methods to improve vertical jump performance. In comparison with local EMS, vibration training or strength training alone, the combination of strength and plyometric training seemed to be the most effective method. It likely took advantage of the enhancement of maximal dynamic force through strength training and the positive effects of ploymetric training on speed and force of muscle contraction through its specific effect on type II fibers and high-threshold alpha-motoneurones. However, the present S+E intervention showed a moderate effect size (SMD) for CODS at 5 m (0.50) and for contact time of DJ (0.63) at posttest. As mentioned before, both interventions, S and S+E, significantly improved the overall performance like the total time of CODS at 15 m and RSI of DJ as well as the height of CMJ and SJ. On the one hand, these results do not suggest negative consequences by the artificial muscle activation by submaximal WB-EMS. EMS does not facilitate learning the specific coordination of complex movement like jumping or sprinting, especially during maximal local isometric stimulation (Perez-Gomez and Calbet, 2013; Paillard, 2018). According to Babault et al. (2007) and Bezerra et al. (2011), a low voluntary movement control exists at maximum stimulation intensities, and only submaximal contractions enable an efficient movement control with superimposed EMS. Perez-Gomez and Calbet (2013) also recommend applying EMS concomitantly with plyometric training or practice of sports. This confirms the approach of the present study by a submaximal WB-EMS superimposed on strength, sprinting, and jumping exercises. On the other hand, the observation of Bezerra et al. (2009) could not be confirmed. VC + EMS caused no additional training effect compared with VC training. They hypothesized that it would activate the same neural pathways that are normally used in voluntary exercise, with additional afferent inputs (centrally integrated) provoked by the electrostimulation.

With a closer look at the results of SSS and the training method EMS, the present dynamic strength training intervention with sprinting and jumping exercises superimposed by submaximal WB-EMS significantly decreased total time (2.3%) of a 30-m linear sprint as well as 5-m (6.3%), 10-m (3.7%), and 20-m (2.9%) split time between pre-, post-, and retests. So far, SSS performance over distances ≥ 30 m could not be improved, neither by the two WB-EMS studies (Filipovic et al., 2016; Wirtz et al., 2016) nor by local EMS studies in male athletes (Herrero et al., 2006, 2010a; Babault et al., 2007; Billot et al., 2010). The diagnostics of force and power parameters as secondary endpoints of this study supported the results of SSS. Superimposed WB-EMS during the Bulgarian split squat and the Nordic curl resulted in increases of 18.2% of biceps femoris force to 12.6% of quadriceps femoris force. According to Mero et al. (1992) and Delecluse (1997), biceps femoris showed the highest EMG activation during the maximum speed of SSS and quadriceps femoris showed the highest EMG activation during the acceleration phase of SSS. Ebben et al. (2009) demonstrated a < 0.5 EMG activation ratio between biceps femoris and quadriceps femoris for the exercises squat and split squat. The 2 dynamic WB-EMS studies used the squat exercise as strength training intervention. Wirtz et al. (2016) applied ALs during superimposed WB-EMS. They showed increases of 8% of biceps femoris force, but they enhanced quadriceps femoris force by 28.6% without a transfer to SSS. Filipovic et al. (2016) used an explosive movement velocity during superimposed WB-EMS. They showed a significant decrease for 5-m split time after 7 weeks. Thus, the acceleration phase was influenced by an improved quadriceps femoris force of 19.9% but not the maximum speed phase of SSS. Moreover, Herrero et al. (2010a) who applied local EMS at quadriceps femoris during knee extension showed no effects on 20-m sprint time, too. This exercise reached 8.6% of maximum VC of biceps femoris instead of 86.9% of quadriceps femoris during EMG analysis (Ebben et al., 2009). On the contrary, three local EMS studies showed positive effects on SSS over distances ≤20 m (0.8–2.3%) (Herrero et al., 2006, 2010b; Voelzke et al., 2012). They used isometric EMS of quadriceps femoris in combination with plyometrics. Plyometrics reached a higher biceps femoris to quadriceps femoris activation ratio of 1.01 in precontact and 0.55 in post-contact (Ebben et al., 2010). Thus, a strength exercise selection superimposed by WB-EMS with a high biceps femoris activation and a positive activation ratio to quadriceps femoris like the Nordic curl appear reasonable to enhance SSS, especially over distances ≥ 30 m. Moreover, in contrast to CODS, SJ or CMJ performance, additional jumping and sprinting exercises or an explosive movement velocity during strength exercises seem to be crucial in the application of EMS or WB-EMS to improve SSS.

Some limitations of the present study have to be mentioned for further research on WB-EMS. Six dropouts, three in each group without a coherent statement of reasons, occurred. Thus, it seemed to be independent of the intervention with or without WB-EMS. The final 22 participants, sufficient according to the *a priori* power analyses, improved strength, power, and jumping performance without a detraining phase. This indicated that strength trained female sport students were able to cope with the physical requirements. In particular, the WB-EMS training at 70% iPT seemed to be a beneficial compromise to achieve strength and power adaptations as well as to have an appropriate exertional tolerance. A detraining period of 2 to 6 weeks is common in several EMS studies (Filipovic et al., 2011). However, this should be further verified with additional physiological parameters like creatine kinase, questionnaires about physical habits, and recovery–stress states. Moreover, a control, blinding, or placebo intervention group could verify the attribution of environmental influences, expectations, or learning effects on performance gains. With reference to Sommi et al. (2018), a broad acceptance of superimposed WB-EMS by female athletes is decisive for further development of athletic programs. Due to the lack of WB-EMS studies in female athletes, the results of this study were often compared with studies in male athletes. Although Maffiuletti et al. (2008) demonstrated that supramotor thresholds were significantly lower in women than in men, contrary to the expected constitutional differences like subcutaneous fat thickness, women showed no significant differences at motor threshold. However, the subjective tolerance to current intensity remains a key limiting factor of EMS, regardless of sexes (Reed, 1997).

Finally, the conclusion of our investigation is that superimposed submaximal WB-EMS during dynamic strength, sprinting, and jumping exercises could serve as a reasonable but not superior alternative to classic training regimes to improve CODS and SSS, VJ and HJ, as well as strength and power parameters in physically active female. The present WB-EMS approach at 70% of iPT seems to intensify dynamic strength exercises equal but not higher than ALs corresponding to the 8 to 10 repetition maximum. Thus, against the hypothesis, it leads to comparable but no greater improvements in physical fitness. Therefore, it remains to be considered whether the effort of a submaximal superimposed WB-EMS short-term intervention is remunerative. Whether it provides perspectives for female athletes with little experiences or insufficient technique to incorporate strength routines without using moderate to high AL, which is necessary to improve sprinting performance or to provide injury prevention and joint stability, has to be verified in further studies. Moreover, training regimes concentrating on contact time of DJ or CODS at 5 m should be executed without superimposed WB-EMS in physically active females concerning to the present results and has to be verified in further WB-EMS studies, too. Additionally, an improvement of SSS performance over a distance of ≥30 m occurs for the first time for superimposed local EMS or WB-EMS. To improve SSS at maximum speed by superimposed WB-EMS, our results offer a combination of jumping and sprinting exercises with strength exercises that have a high biceps femoris activation and a positive activation ratio to quadriceps femoris. In this context, a higher transferability of physically active females than males and the adaptations of WB-EMS over time need to be further researched, as well as concepts for periodization in high-performance sports need to be developed.

## Ethics Statement

The study protocol was approved by the “Ethics Committee of the German Sport University Cologne” in December 2013 and complied with the Declaration of Helsinki.

## Author Contributions

UD, NW, and FM conceived and designed the research. UD, NW, FM, and MM conducted the experiments. UD analyzed the data and wrote the manuscript. HK, NW, FM, and LD revised the manuscript. All authors read and approved the manuscript.

## Conflict of Interest Statement

The authors declare that the research was conducted in the absence of any commercial or financial relationships that could be construed as a potential conflict of interest.
