# Supplementary material for: The Effects of Superimposed Whole-Body Electromyostimulation During Short-Term Strength Training on Physical Fitness in Physically Active Females: A Randomized Controlled Trial
Source: Front Physiol. 2019 Jun 27;10:728. doi: 10.3389/fphys.2019.00728 (PMC6610316; doi:10.3389/fphys.2019.00728)
Supplement: Supplementary file 1 [file Data_Sheet_1.pdf]

frontiers

Supplementary Material

The Effects of Superimposed Whole-Body Electromyostimulation During Short-Term Strength Training on Physical Fitness in Physically Active Females: A Randomized Controlled Trial.

Ulrike Dörmann[1], Nicolas Wirtz[1], Florian Micke[1], Mareike Morat[1], Heinz Kleinöder[1], Lars Donath[1]*

[1]Institute of Training Science and Sport Informatics, Department of Intervention Research in Exercise Training, German Sport University Cologne, Cologne, Germany.

***Correspondence:**
Prof. Dr. Lars Donath, PhD
Department of Intervention Research in Exercise Training
Am Sportpark Müngersdorf 6, 50933 Cologne, Germany
l.donath@dshs-koeln.de
Phone: +49 221 4982 7700

1 Supplementary Figure

Supplementary Figure S1. WB-EMS device and bilaterally paired surface electrodes: stimulation vest and electrical cords (on the left side), EMS device and electrodes for the lower legs and the thighs (in the middle) and the electrodes for the buttocks (on the right side).

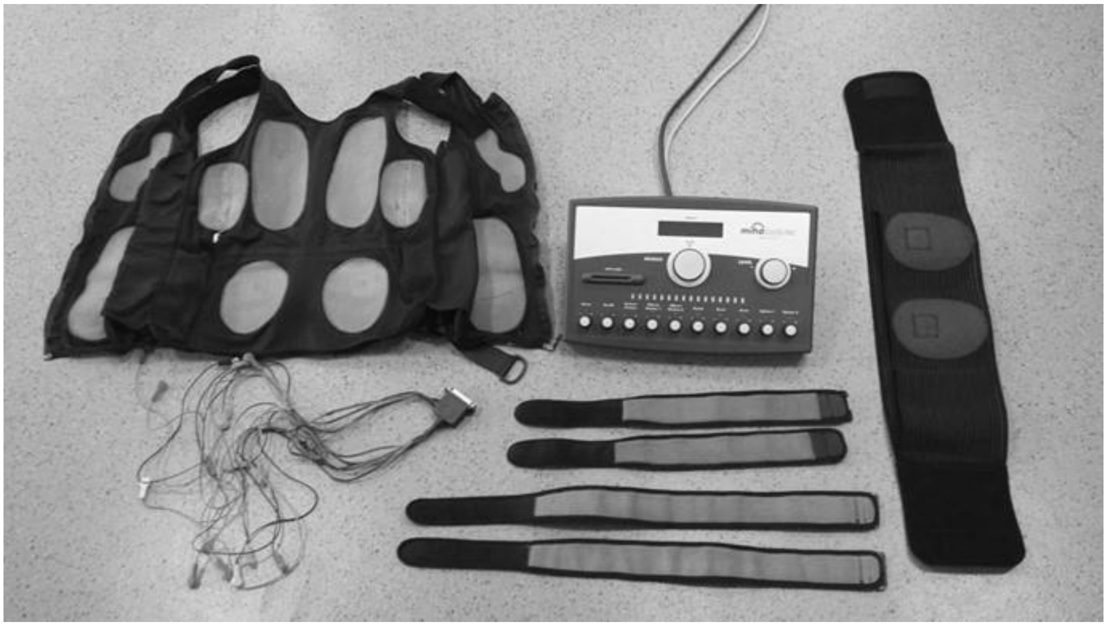

2 Supplementary Tables

Supplementary Table S1. Strength Training Exercises.

Strength Training Exercises	
(1) Bulgarian Split Squad	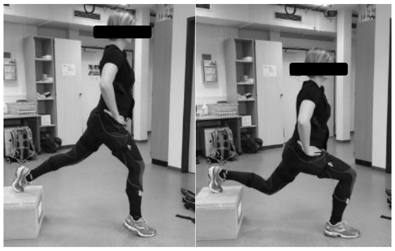
(2) Nordic Curl	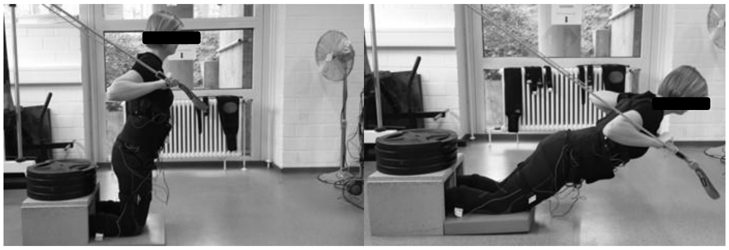
(3) Knee Tuck	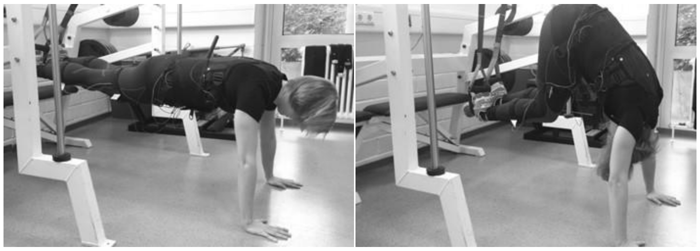
(4) Side Abs	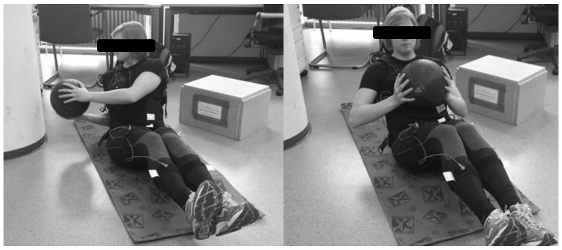

Supplementary Table S2. Sprinting and Jumping Exercises.

**Sprinting and Jumping Exercises**	
(1) Skipping	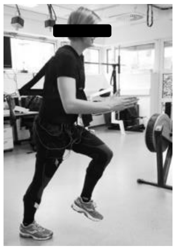
(2) Heeling	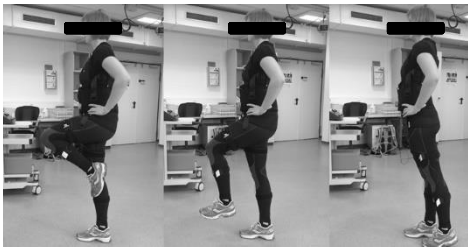
(3) Side Jumps	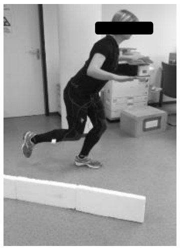
(4) Box Jumps	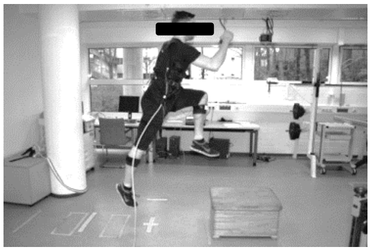

(5) Drop Jumps	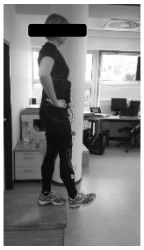
